# Acid sphingomyelinase activity is regulated by membrane lipids and facilitates cholesterol transfer by NPC2[S]

**DOI:** 10.1194/jlr.M054528

**Published:** 2014-12

**Authors:** Vincent O. Oninla, Bernadette Breiden, Jonathan O. Babalola, Konrad Sandhoff

**Affiliations:** * LIMES Institute, Membrane Biology and Lipid Biochemistry Unit, Kekulé-Institut für Organische Chemie und Biochemie, Universität Bonn, D-53121 Bonn, Germany; † Department of Chemistry, University of Ibadan, Ibadan, Nigeria

**Keywords:** Niemann-Pick protein type C2, sphingomyelin, phosphatidylcholine, anionic phospholipids, ceramide, diacylglycerol, cationic lipids

## Abstract

During endocytosis, membrane components move to intraluminal vesicles of the endolysosomal compartment for digestion. At the late endosomes, cholesterol is sorted out mainly by two sterol-binding proteins, Niemann-Pick protein type C (NPC)1 and NPC2. To study the NPC2-mediated intervesicular cholesterol transfer, we developed a liposomal assay system. (Abdul-Hammed, M., B. Breiden, M. A. Adebayo, J. O. Babalola, G. Schwarzmann, and K. Sandhoff. 2010. Role of endosomal membrane lipids and NPC2 in cholesterol transfer and membrane fusion. *J. Lipid Res*. 51: 1747–1760.) Anionic lipids stimulate cholesterol transfer between liposomes while SM inhibits it, even in the presence of anionic bis(monoacylglycero)phosphate (BMP). Preincubation of vesicles containing SM with acid sphingomyelinase (ASM) (SM phosphodiesterase, EC 3.1.4.12) results in hydrolysis of SM to ceramide (Cer), which enhances cholesterol transfer. Besides SM, ASM also cleaves liposomal phosphatidylcholine. Anionic phospholipids derived from the plasma membrane (phosphatidylglycerol and phosphatidic acid) stimulate SM and phosphatidylcholine hydrolysis by ASM more effectively than BMP, which is generated during endocytosis. ASM-mediated hydrolysis of liposomal SM was also stimulated by incorporation of diacylglycerol (DAG), Cer, and free fatty acids into the liposomal membranes. Conversely, phosphatidylcholine hydrolysis was inhibited by incorporation of cholesterol, Cer, DAG, monoacylglycerol, and fatty acids. Our data suggest that SM degradation by ASM is required for physiological secretion of cholesterol from the late endosomal compartment, and is a key regulator of endolysosomal lipid digestion.

Macromolecules and membrane components of mammalian cells move to lysosomes for digestion by autophagy, phagocytosis, and endocytotic pathways. Their building blocks are released as nutrients into the cytosol. Complex lipids of the plasma membrane end up in the luminal intraendolysosomal vesicles, which serve as platforms for their digestion by water-soluble hydrolases with the essential help of lysosomal lipid binding and transfer proteins. Inherited deficiencies of these proteins cause fatal lipid and membrane storage diseases (1).

During endocytosis, a lipid sorting process adjusts the luminal vesicles and inner endosomal membranes for digestion (2, 3). Undegradable cholesterol is sorted out by two cholesterol binding proteins, Niemann-Pick protein type C (NPC)2 and NPC1 (4–8). Their inherited deficiencies lead to Niemann-Pick disease type C. NPC2 can pick up cholesterol directly from inner membranes and transfer it to NPC1 or other vesicular membranes. The intervesicular transfer of cholesterol by NPC2 is regulated by the lipid composition of the vesicular membranes (7, 9).

Anionic lipids, like bis(monoacylglycero)phosphate (BMP), stimulate the NPC2-mediated cholesterol transfer which is increased in the presence of ceramide (Cer), whereas the plasma membrane-derived SM inhibits cholesterol transfer even in the presence of BMP (7). This suggests that acid sphingomyelinase (ASM) (SM phosphodiesterase, EC 3.1.4.12) plays a role in the secretion of cholesterol from the luminal vesicles.

Previously, we have purified homogeneous human ASM to generate peptides for sequencing, isolation of cDNAs, and analysis of its genomic structure (10–13). This aided the identification of the molecular basis of Niemann-Pick disease types A and B (14, 15). This disease is characterized by an endolysosomal accumulation of SM, and secondarily by unesterified cholesterol and BMP (16–18).

Mature endolysosomal ASM is generated as a highly glycosylated 70 kDa protein from a precursor protein through the proteolytic process. The biosynthesis of the 75 kDa ASM prepro-protein is followed by a stepwise proteolytic process, generating a highly glycosylated 72 kDa active precursor protein inside the endoplasmic reticulum and Golgi complex, and an enzymatically active mature 70 kDa enzyme in the late endosomes/lysosomes. In fibroblasts, the latter is further split to an inactive 52 kDa form (19–21). The mature form consists of an N-terminal intramolecular saposin (Sap) domain (22), which has the same disulfide bound pattern as Saps (23), a proline-rich domain, a metallophosphoesterase/catalytic domain, and a C-terminal domain (24). In addition to the lysosomal enzymatically active form, a secretory form of ASM exists; both are generated from the same gene by differential splicing and posttranslational modification, and are transported by alternative pathways (21, 24). Both precursor and secreted forms of ASM are enzymatically active. The lysosomal form of cultured human fibroblasts is sensitive to cationic amphiphilic drugs. Cationic amphiphilic drugs ingested by cultured fibroblasts can reach the luminal vesicles of endosomes and lysosomes where they compensate for their negative surface charge, thereby releasing the cationic ASM and other cationic hydrolases from the vesicular surfaces. The released enzymes are then degraded (25, 26) by lysosomal proteases, e.g., cathepsin B (27, 28).

Several lysosomal sphingolipid hydrolases require sphingolipid activator proteins (Sap A, Sap B, Sap C, Sap D, or GM2 activator protein) as cofactors for the in vivo degradation of glycosphingolipids with short hydrophilic headgroups (29). The in vivo degradation of SM, however, does not require an activator protein for physiological turnover, because patients deficient in prosaposin do not accumulate SM (30) due to unchanged ASM activity (31, 32).

The mechanisms controlling the digestion of SM are not fully understood. The in vivo and in vitro activity of ASM against lipid substrates is regulated by several factors like pH, lipid transfer-proteins (e.g., Sap C) (10, 24, 33, 34), and the lipid composition of the substrate-carrying membranes (see below). It has been demonstrated that phosphatidylinositol (PI)-phosphates PI(3,4,5)P_3_ and PI(4,5)P_2_ (both located at the plasma membrane), and PI(3,5)P_2_ (perimeter membrane of late endosome/lysosome) inhibit ASM activity (10, 35, 36), whereas lipids like BMP and PI, which are enriched in the intravesicular membranes of the late endosome/lysosome (37, 38), stimulate the digestion of SM (34). Earlier studies using detergent-based and nonliposomal assays also indicated that phosphatidic acid (PA), phosphatidylcholine (PC), unsaturated tri-, di-, and monoacylglyceride, cholesterol, and free fatty acids, as well as Cer, can enhance the hydrolysis of SM under appropriate conditions (10, 39).

Homogeneous human ASM with a specific activity of 2.5 mmol SM split/h/mg ASM protein was originally purified using a detergent-based micellar assay (10). In the present study, we used a detergent-free liposomal assay to study the action of recombinant human ASM on membrane bound radiolabeled lipids. The liposomes carrying the radiolabeled phospholipid mimic the luminal endolysosomal vesicles, which accumulate in the skin cells of patients with an inherited prosaposin deficiency (31). ASM was identified as an unspecific phospholipase C, digesting also PC, the activity (and specificity) of which is regulated by the lipid composition of the substrate carrying liposomal membranes. The obtained results suggest a sequence of events involved in the maturation of luminal vesicles in the late endosomes. ASM digests SM of the luminal vesicles, thereby cancelling the inhibitory role of SM on cholesterol secretion by NPC2 in the late endosomes.

## MATERIALS AND METHODS

### Materials

Dioleoyl-PC (DO-PC), bis(monooleoylglycero)phosphate (BMP), 1,2-dioleoyl-*sn*-glycero-3-ethylphosphocholine (EPC), *N*1-[2-((1S)-1-[(3-aminopropyl)amino]4-[di(3-amino-propyl)amino] butylcarboxamido)ethyl]3,4-di[oleyloxy]benzamide [multivalent cationic lipid (MVL5)], and 1,2-di-*O*-octadecenyl-3-trimethylammonium propane (DOTMA) were obtained from Avanti Polar Lipids (Alabaster, AL). Cholesterol, dipalmitoyl-PC, diarachidonoyl-PC, oleoylstearoyl-PC, 1-palmitoyl-2-arachidonoyl-PC, dipalmitoyl-PA, dipalmitoyl-phosphatidylserine (PS), dipalmitoyl-phosphatidyl­glycerol (PG), PI, 1,2-dipalmitoylglycerol, 1-stearoylglycerol, lyso-PC, and dihexadecylphosphate (DHP) (also called dicetylphosphate) were purchased from Sigma (Taufkirchen, Germany). C18-SM was purchased from Matreya (Pleasant Gap, PA) and 1-oleoyl-2-palmitoyl-PC from Supelco (Bellefonte, PA). Distearoyl-PC and palmitic acid were obtained from Fluka (Buchs, Switzerland). D-*erythro*-C18-Cer and [choline methyl-^3^H]SM ([^3^H]SM) (70 Ci/mol) were available in our lab. The radiolabeled lipid [1-^14^C]stearoyl-SM ([^14^C]SM) (55 Ci/mol) was obtained from American Radiolabeled Chemistry (St. Louis, MO). 1-[^14^C]dipalmitoyl-L-α-PC ([^14^C]PC) (110 Ci/mol) was from PerkinElmer (Boston, MA) and 4-[^14^C]cholesterol (58 Ci/mol) from GE Healthcare (Buckinghamshire, UK).

*N*-(7-nitrobenz-2-oxa-1,3-diazol-4-yl)-1,2-dihexadecanoyl-*sn*-glycero-3-phosphoethanolamine triethyl ammonium salt (NBD-PE) and *N*-[6-(biotinoyl)amino]hexanoyl-1,2-dihexadecanoyl-*sn*-glycerol-3-phosphoethanolamine triethyl ammonium salt (Biotin-PE) were purchased from Invitrogen Molecular Probes (Eugene, OR). BioMag streptavidin suspension (5 mg/ml) and the magnetic separation stand, MagneSphere, were obtained from Qiagen (Hilden, Germany) and Promega (Madison, WI), respectively. All other chemicals were obtained from Sigma-Aldrich and Merck. Chemicals and solvents were of analytical grade.

### Protein preparation

Recombinant human ASM was expressed in Sf21 insect cells using the baculovirus expression vector system (40, 41). The specific activity of the ASM used in this study, as determined in a micellar detergent-based assay system (10, 35), was 148 μmol/h/mg.

Bovine NPC2 was isolated from milk and purified as previously described (42).

### Micellar ASM activity assay

The micellar detergent-based assay was performed as previously described (10, 35).

Assay mixtures contained 250 mM sodium-acetate buffer (pH 4.5), 0.1% (w/v) Nonidet P-40, 3.8 ng purified ASM, and a mixture of unlabeled SM and [^3^H]SM (labeled at the methyl residue of the choline group) with a final SM concentration of 100 μM/50 μl assay volume. The assay was incubated at 37°C for a time period of 0–120 min. The reactions were terminated by adding 800 μl chloroform/methanol 2/1 (v/v) and 250 μl water. The upper aqueous layer was separated from the organic layer by gentle vortexing and centrifugation at 12,000 *g*. Liberated [^3^H]phosphorylcholine was quantified in a 200 μl aliquot from the upper phase using a Tricarb 2900TR liquid scintillation analyzer (PerkinElmer, Rodgau, Germany). One unit of enzymatic activity is defined as the amount of enzyme that catalyzes the hydrolysis of 1 mmol SM per hour in the detergent-based assay system.

### Preparation of liposomes

Liposomes were prepared as previously described (43). Unless stated otherwise, neutral liposomes contained 80 mol% PC (77 mol% DO-PC and 3 mol% [^14^C]PC), 10 mol% cholesterol, and 10 mol% SM (9.5 mol% SM and 0.5 mol% [^14^C]SM). Negatively or positively charged liposomes generally consisted of 60 mol% PC (57 mol% DO-PC and 3 mol% [^14^C]PC), 20 mol% anionic lipid (BMP, PA, PG, PI, PS, or DHP) or cationic lipid (EPC, MVL5, DOMTA), 10 mol% cholesterol, and 10 mol% SM (9.5 mol% SM and 0.5 mol% [^14^C]SM). When the composition of liposomes was varied, the appropriate amount of DO-PC was replaced, while the mole percent of all other components was kept constant. Liposomes labeled with [^14^C]cholesterol contained 10 mol% unlabeled SM.

Vesicles were prepared by mixing appropriate amounts of lipids from stock solutions and evaporating the solvents under a stream of nitrogen. The lipid mixture was hydrated in 20 mM citrate buffer (pH 5.0), containing 150 mM NaCl to a final lipid concentration of 200 nmol/ml. The dispersion was vortexed, sonicated in a Branson sonifier (Danbury, CT) at 120 W for 30 s, and subjected to eight freeze-thaw cycles. Thereafter, large unilamellar vesicles were prepared by extrusion through polycarbonate filters with a pore size of 100 nm mounted in tandem in a mini-extruder (LiposoFast, Avestin, Ottawa, Canada). Samples were subjected to 21 passes.

The sizes of the liposomes were checked using dynamic light scattering (DLS). The size distribution measurements were performed with an ALV-NIBS high-performance particle sizer (ALV, Langen, Germany) operated at a wavelength of 632.8 nm and with a detection angle of 173°. For each measurement, 400 μl liposome solution was used at 25°C.

The surface charges of the liposomes were analyzed using the determination of the zeta (ζ) potential using Zetasizer Nano ZS (Malvern Instruments, Malvern, UK). The experiments were performed at 25°C in 20 mM citrate buffer (pH 5.0).

### Liposomal ASM assay

The liposomal assay mixture for determination of ASM activity contained 40 μl (8 nmol total lipid) of a liposomal preparation and, unless stated otherwise, 150 ng purified ASM in 20 mM sodium citrate buffer containing 150 mM sodium chloride (pH 5.0) in a final volume of 50 μl. After 2 h of incubation at 37°C, the reaction was stopped by the addition of 140 μl chloroform/methanol 2/5 (v/v), to obtain a homogeneous phase. Lipids were dried under a stream of nitrogen and redissolved with 60 μl chloroform/methanol 1/1 (v/v) and vortexed. The lipids were separated sequentially up to 7.5 cm with chloroform/methanol/water 60/25/4 (v/v/v) and up to 10 cm with n-hexane/diethyl­ether/glacial acetic acid 70/30/1 (v/v/v) by thin-layer chromatography using a high-performance thin-layer chromatography plate (Merck, Darmstadt, Germany). Radioactive bands were visualized with a Bio Imaging Analyzer 1000 (Fuji, Japan), and the quantification was performed with the image analysis software “Tina” (Raytest, Staubenhardt, Germany).

### Cholesterol transfer assay

To investigate cholesterol transfer in the presence of ASM, 4 nmol of donor vesicles {9 mol% cholesterol, 1 mol% [^14^C]cholesterol, 10 mol% SM, 20 mol% BMP, 4 mol% Biotin-PE, and 56 mol% DO-PC in 20 mM citrate buffer (pH 5.0), containing 150 mM NaCl; 200 nmol final lipid/ml} and 20 nmol acceptor liposomes [10 mol% cholesterol, 10 mol% SM, 20 mol% BMP, 4 mol% NBD-PE, and 56 mol% DO-PC in 20 mM citrate buffer (pH 5.0), containing 150 mM NaCl; 1,000 nmol final lipid/ml] were preincubated with ASM for a period of 0–110 min at 37°C in a final volume of 200 μl. Thereafter, 0.1 nmol NPC2 was added and incubated further for 10 min at 25°C. Lipid transfer was stopped by adding 75 μl of 1 M Tris buffer (pH 8). To separate the donor and acceptor vesicles, streptavidin-coated beads were used. Aliquots of the supernatant were measured for radioactivity and fluorescence (to control the recovery). The calculations were performed as previously described (7).

To monitor the conversion of SM to Cer by ASM, a parallel experiment was performed using donor liposomes which contained [^14^C]SM and unlabeled cholesterol. The assay was stopped with 140 μl chloroform/methanol 2/5 (v/v) and the lipids were dried under a stream of N_2_. SM and Cer were separated with chloroform/methanol/water 60/25/4 (v/v/v) by thin-layer chromatography, and the amounts were quantified using a phosphoimaging analyzer (Fuji, Japan).

### Surface plasmon resonance spectroscopy

Real-time interaction analysis between proteins and immobilized liposomes was performed using surface plasmon resonance spectroscopy at 25°C in a Biacore® 3000 instrument (Biacore, now GE Healthcare). Liposomes were immobilized on the surface of a Pioneer® L1 sensor chip (GE Healthcare) until saturation, as indicated by a constant signal [response units (RU) of cationic liposomes, ∼15,000 RU; neutral liposomes, 7,000–9,000 RU; anionic liposomes, 6,000–8,000 RU; PI-containing liposomes, 4,000 RU). For immobilization, 60 μl of 0.5 mM liposomes in 20 mM citrate buffer (pH 4.24) were injected at a flow rate of 5 μl/min. Unbound liposomes were removed by washing with 10 μl buffer at the same flow rate. To ensure a complete saturation of the surface, an additional 20 μl of the liposome solution was injected and the surface was washed with 10 μl buffer at a flow rate of 100 μl/min to stabilize the baseline. At this stage, the signal was adjusted to zero. Thereafter, the ASM [40 μg/ml in 20 mM citrate buffer (pH 4.24)] was injected at a flow rate of 20 μl/min for 180 s (association phase), followed by buffer only for 180 s (dissociation phase).

### Fusion assay

The fusion assay and the calculation were performed as previously described (7).

## RESULTS

### ASM stimulates the NPC2-mediated cholesterol transfer between liposomal vesicles

During endocytosis, the digestion of complex membrane lipids and lipid sorting take place at luminal vesicles and inner membranes of the late endosomes/lysosomes. Cholesterol is sorted out mainly by two cholesterol binding proteins, the soluble NPC2 and NPC1, the latter being a transmembrane protein in the limiting endosomal membrane. The NPC2-mediated cholesterol transfer is regulated by the lipid composition of the intraendosomal membranes. In a previous study, we have shown that the cholesterol transfer by NPC2 is stimulated by the anionic lipid BMP, and much more in the presence of both BMP and Cer; whereas, it is inhibited by SM, even in the presence of BMP (7). These results indicate that ASM, which hydrolyzes SM to Cer, plays an important role in the export of cholesterol from the intraendolysosomal membranes by NPC2. This view is consistent with the observation that Niemann-Pick disease type A and type B patients, which have a profound reduction of ASM activity, not only show SM storage, but also show an accumulation of cholesterol (17). To test this hypothesis, SM- and cholesterol-containing liposomes were preincubated with ASM for different periods of time (0–110 min). Then, NPC2 was added to the mixture of preincubated donor and acceptor vesicles and incubated for a further 10 min. The cholesterol transfer was analyzed as previously described (7, 44). In a parallel experiment, the conversion of SM to Cer by ASM using donor liposomes containing [^14^C]SM and unlabeled cholesterol were analyzed.

**Fig. 1.**
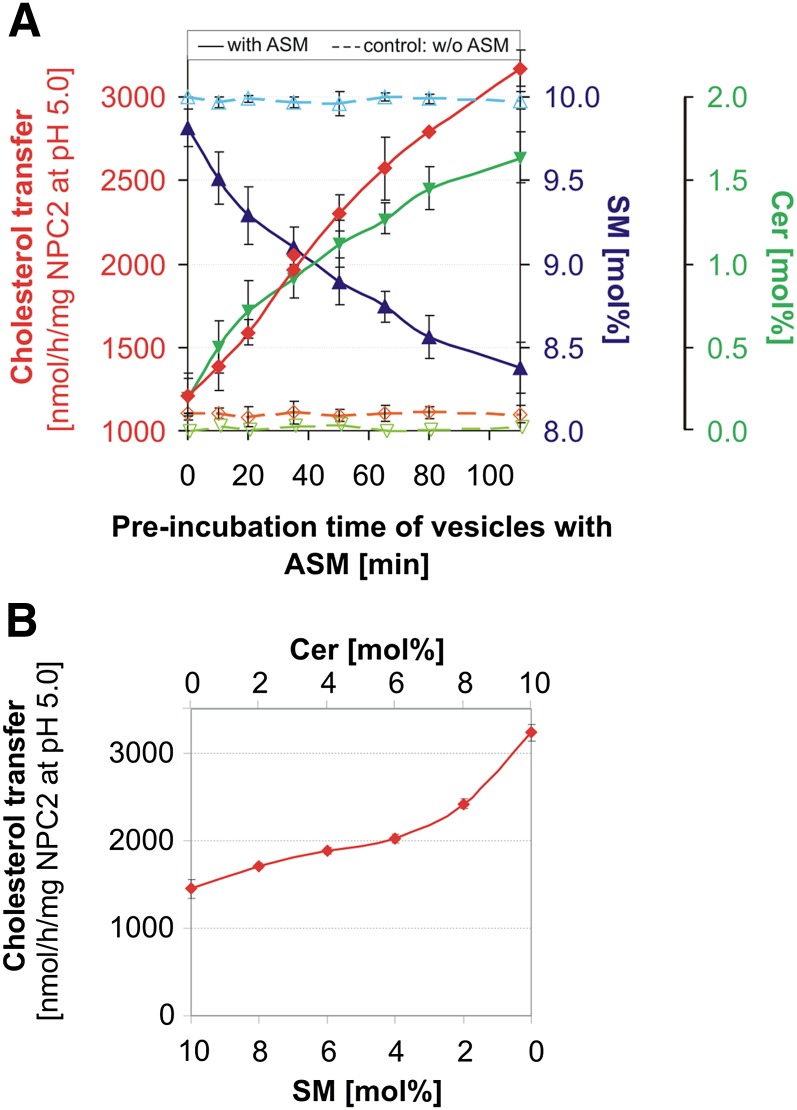
Degradation of SM by ASM stimulates intervesicular cholesterol transfer by NPC2. A: Anionic donor and acceptor vesicles were mixed in a ratio of 1 to 6. The liposomes contained 10 mol% cholesterol, 10 mol% SM, 20 mol% BMP, 56 mol% PC, and 4 mol% Biotin-PE (donor) or NBD-PE (acceptor), respectively. Donor liposomes containing [^14^C]cholesterol were used for the determination of the cholesterol transfer, and in a parallel experiment [^14^C]SM-containing donor liposomes were used to measure the conversion of SM to Cer. Incubation mixtures containing donor and acceptor liposomes with a volume of 200 μl each were preincubated with 200 ng ASM for 0–110 min at 37°C. Thereafter, 0.1 nmol NPC2 was added and the mixture was incubated for a further 10 min at 25°C. After treatment with ASM and NPC2, vesicles were assayed for cholesterol transfer (red), SM content (blue), and Cer content (green). As a control, the samples were incubated without ASM (dashed lines, cholesterol transfer in orange, SM content in light blue, and Cer content in light green). Error bars indicate SEM (n = 4). B: NPC2-mediated cholesterol transfer is enhanced by increasing Cer (0–10 mol%) and decreasing SM (10–0 mol%) levels in the absence of ASM. Error bars indicate SEM (n = 2).

As presented in **Fig. 1A**, SM content decreased and Cer content increased with increasing preincubation periods. Simultaneously, NPC2-mediated cholesterol transfer increased from donor to acceptor vesicles. After a preincubation period of 110 min, the cholesterol transfer rose from 1.2 to 3.2 μmol/h/mg NPC2. During the incubation with ASM, the SM content of the liposomes dropped from 10 to 8.4 mol% and the Cer content increased from 0 to 1.6 mol%. This suggests that about 30% of SM in the outer leaflet of the liposomes is degraded to Cer. Control experiments in the absence of ASM showed no changes, neither in the SM content nor in the cholesterol transfer rate. Control experiments in the absence of NPC2 yielded a small cholesterol transfer that was not protein-mediated, in the range of 150–300 pmol/h, as observed previously (7). This rate was not affected by the absence or presence of ASM (data not shown). As outlined in Fig. 1B, experiments with fixed SM (in the range of 10–0 mol%) and Cer (0–10 mol%) concentrations in the absence of ASM, however, resulted in lower cholesterol transfer rates than given in Fig. 1A.

Because protein-mediated membrane fusion could also distort the results of the transfer assay, we investigated the ability of ASM to induce membrane fusion under the experimental conditions used, by employing a fusion assay (7) and DLS. Neither membrane fusion nor changes of the size of liposomes were observed during ASM incubation (data not shown).

### Liposomal assay to investigate the influence of different membrane lipids on ASM activity

Previous reports have shown that during partial preparation/purification of ASM from human tissue, SM and also some glycerophospholipids like PC and PG were hydrolyzed at a low rate (45, 46). This was also demonstrated with a highly purified ASM preparation (10). To study substrate specificity and regulation of ASM, a detergent-free liposomal assay was developed to investigate the influence of different membrane lipids and their hydrolysis products on the activity of a recombinant human ASM (purified to homogeneity) (40, 41) toward membrane-bound SM and PC. To mimic the inner membranes of the late endosomes, liposomes with an average diameter of 100–140 nm containing 10 mol% SM and 10 mol% cholesterol, in absence or presence of 20 mol% anionic lipid, were used with DO-PC as the host lipid. The liposomes used contained, for the simultaneous analysis of SM and PC degradation within the same assay, 0.5 mol% fatty acid-labeled [^14^C]SM and 3 mol% fatty acid-labeled [^14^C]PC. After incubation, SM and PC and their resulting hydrolysis products, Cer and diacylglycerol (DAG), were separated by thin-layer chromatography and quantified.

### Acyl chain composition of the host lipids strongly affects the cleavage of SM and PC in the liposomal assay

**Fig. 2.**
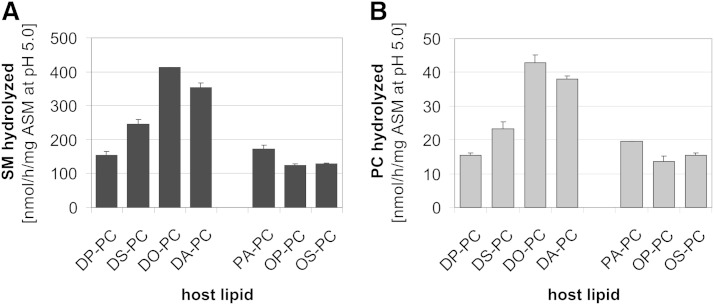
Host lipids carrying unsaturated acyl chains stimulate cleavage of SM and PC by ASM in anionic liposomes. The hydrolysis of SM (A) and PC (B) by ASM was measured simultaneously in the same detergent-free liposomal assay. Liposomes contained 60 mol% PC as a host lipid, 20 mol% PA, 10 mol% cholesterol, and 10 mol% SM. Total lipids (8 nmol) were incubated with 250 ng recombinant ASM in 20 mM sodium citrate buffer containing 150 mM sodium chloride (pH 5.0) in a final volume of 50 μl for 120 min at 37°C. The formation of Cer and DAG was analyzed by quantification using thin-layer chromatography. DP-PC, dipalmitoyl-PC; DS-PC, distearoyl-PC; DA-PC, diarachidonoyl-PC; PA-PC, 1-palmitoyl-2-arachidonoyl-PC; OP-PC, oleoylpalmitoyl-PC; OS-PC, oleoylstearoyl-PC. Error bars indicate SEM (n = 4).

At first, the effect of acyl chain length and saturation of host lipids on the SM and PC degradation by ASM (**Fig. 2**) was analyzed to optimize the assay conditions. Digestion of both lipids was higher in liposomal membranes containing unsaturated PC species, rather than mixed or saturated PC species. In DO-PC-containing liposomes, the highest ASM activity measured for hydrolysis of SM was 413 nmol/h/mg ASM and 43 nmol/h/mg ASM for PC. The hydrolysis rate followed the order: DO-PC > diarachidonoyl-PC (∼88% of DO-PC) > distearoyl-PC (∼55% of DO-PC) > dipalmitoyl-PC (∼35% of DO-PC). For the following experiments DO-PC was used as the membrane-forming host lipid.

Irrespective of the PC species used, ASM-catalyzed hydrolysis of SM was always 10- to 12-fold faster than PC.

### Anionic lipids stimulate SM degradation by ASM

**Fig. 3.**
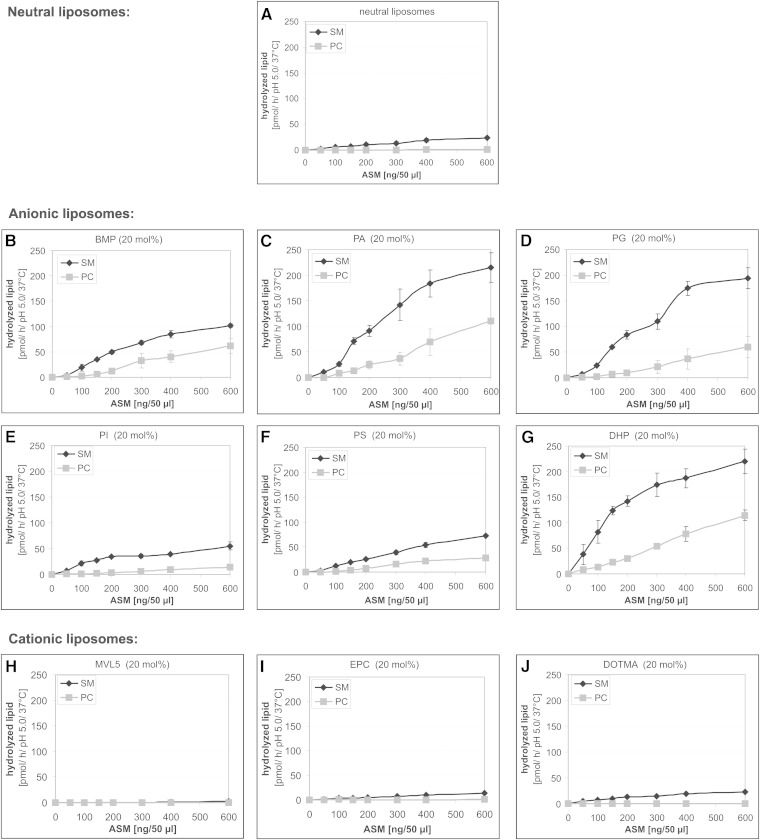
Anionic membrane lipids stimulate the cleavage of liposomal SM and PC by ASM. Hydrolysis of SM (black line) and PC (gray line) was determined simultaneously in the same assay at pH 5.0 by variation of the ASM concentration in neutrally, anionically, and cationically charged liposomes. Neutral liposomes (A) contained 80 mol% DO-PC, 10 mol% cholesterol, and 10 mol% SM. Negatively charged liposomes generally consisted of 60 mol% DO-PC, 20 mol% anionic lipid [BMP (B), PA (C), PG (D), PI (E), PS (F), and DHP (G)], 10 mol% cholesterol, and 10 mol% SM. Cationic vesicles contained 20 mol% cationic lipid [MVL5 (H), EPC (I), DOMTA (J) instead of the anionic lipids]. Error bars indicate SEM (n = 4).

The influence of several anionic phospholipids occurring in cytosolic leaflets of cellular membranes (PA, PG, PI, and PS) and of BMP generated in luminal vesicles as a breakdown product of PG on the ASM-mediated cleavage of SM and its glycerolipid analog PC in a liposomal assay system was studied. In addition, the roles of the synthetic anionic lipid, DHP, and those of synthetic cationic lipids (EPC, DOTMA, and MVL5) were investigated. In neutral liposomes, that is, in the absence of anionic and cationic lipids, only small amounts of SM and hardly any PC were cleaved by ASM (SM was 51.07 ± 3.58 nmol/h/mg ASM and that of PC was 2.50 ± 0.37 nmol/h/mg) (**Fig. 3A**). In the presence of anionic membrane lipids, the rate of ASM-catalyzed SM and PC hydrolysis increased substantially (Fig. 3B–G). Anionic phospholipids derived from the plasma membrane, PA and PG (Fig. 3C, D), were more effective stimulators than BMP, which is generated at the inner membranes of the late endosomes/lysosomes (Fig. 3B). On the other hand, PI and PS were less effective (Fig. 3E, F). The ASM-mediated hydrolysis rate of liposomal phospholipids increased in the presence of PA up to 467 ± 8 nmol SM/h/mg and 140 ± 22 nmol PC/h/mg. The stimulatory effect on the SM digestion varied with the nature of the anionic phospholipid used: PA (8.5-fold) > PG (8.1-fold) > BMP (4.4-fold) > PS (2.9-fold) > PI (2.3-fold). Compared with SM, the hydrolysis rate of PC increased much more in the presence of naturally occurring anionic phospholipids [PA (56.0-fold) > BMP (33.4-fold) > PG (28.2-fold) > PS (17.2-fold) > PI (8.2-fold)]. The stereochemistry of BMP (S/S, R/R, R/S stereoisomers) had no significant effect on the rate of SM and PC degradation by ASM (data not shown), whereas the synthetic phospholipid, DHP, was as effective as PA and PG (Fig. 3G). On the other hand, cationic lipids inhibited ASM activity. MVL5, which has five positive charges on its headgroup at low pH values, had the strongest inhibitory effect (Fig. 3H, supplementary Fig. IB). EPC reduced the ASM activity by half (Fig. 3I, supplementary Fig. IC), whereas DOTMA had no significant effect on the cleavage of SM and PC (Fig. 3J, supplementary Fig. ID). Its positive charge is more localized in the lipophilic part of the membrane and is presumably protected under the headgroups of the neighboring PC and SM molecules, which are zwitterionic and have no net charge.

It seems that electrostatic interaction is very important for the association between cationic ASM and different anionic membranes at endolysosomal pH values in the range of 4.2–5.0. The net charge of ASM and of different liposomes at pH 5.0 was analyzed using ζ potential measurements. The ζ potential of ASM in 20 mM citrate buffer at pH 5.0 was 4.39 ± 0.21 mV, indicating a positively charged protein. Under the same conditions, liposomes containing 20 mol% anionic lipid have negative surface charges (PA, −24.48 ± 1.98 mV; PG, −24.65 ± 1.05 mV; BMP, −26.53 ± 0.36 mV; PS, −23.58 ± 1.36 mV; PI, −21.50 ± 4.33 mV; and DHP, −22.45 ± 1.65 mV). The ζ potential of neutral liposomes was 0.11 ± 1.42 mV and those of cationic liposomes were 23.44 ± 2.17 mV (EPC) and 22.20 ± 1.93 mV (DOTMA).

**Fig. 4.**
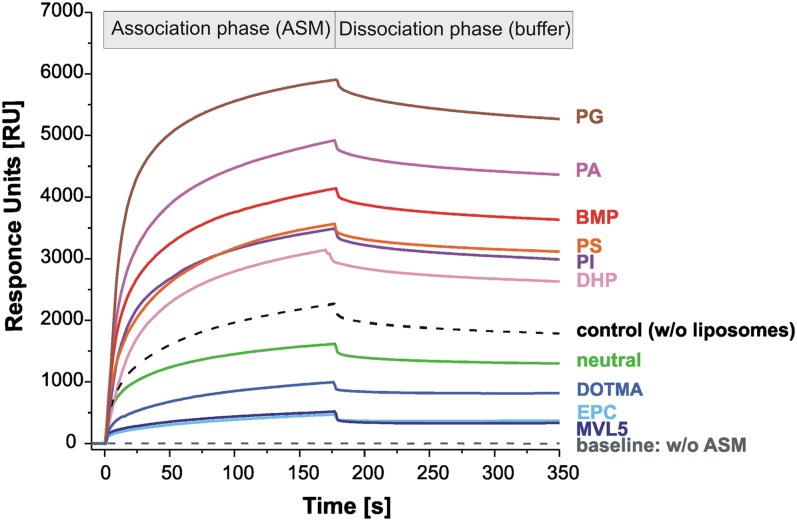
Binding of ASM to differently charged liposomes immobilized on the surface of a L1 sensor chip. Neutral liposomes (green) contained 80 mol% DO-PC, 10 mol% cholesterol, and 10 mol% SM. Negatively charged liposomes generally consisted of 60 mol% DO-PC, 20 mol% anionic lipid (in different red shades), 10 mol% cholesterol, and 10 mol% SM. Cationic vesicles contained 20 mol% cationic lipid (in three blue shades) instead of the anionic lipid. After immobilization of the liposomes on the surface of the L1 sensor chip, ASM in 20 mM citrate buffer (pH 4.24) was injected for 180 s (association phase). Thereafter the chip was washed only with buffer (dissociation phase). The signal of the immobilized liposomes was adjusted to zero. ASM interaction with the L1 chip in the absence of immobilized liposomes (control, dashed black line). Dashed gray line is the baseline, without ASM. Its decrease below the baseline would suggest mobilization of liposomes without any injection of ASM. The difference between the RU value before ASM injection and after the dissociation phase was taken as the amount of ASM bound to the liposomes on the chip.

Previous studies using surface plasmon resonance spectroscopy (Biacore) suggest that ASM binds strongly to BMP-containing membranes (25, 34). Therefore, the relative binding of ASM to positively, negatively, or neutrally charged vesicles was analyzed. A representative sensorgram for each kind of liposome is shown in **Fig. 4**. The binding of ASM to immobilized liposomes is dependent on the ζ potential of the used liposomes. A strong binding of ASM was observed with negatively charged liposomes (2,640–5,280 RU, lines in different red shades) compared with neutral (1,300 RU, green line) and cationic liposomes (345–820 RU, lines in blue shades). As a control experiment, ASM was directly immobilized to the L1 chip (black dashed line). The sensorgram in Fig. 4 shows that ASM binds more strongly to liposomes containing anionic phospholipids (lines in different red shades) than to the free chip (black dashed line) or to neutral (green line) and cationic liposomes (lines in blue shades).

### Cholesterol affects SM and PC hydrolysis by ASM differently

As previously shown (7) and demonstrated in Fig. 1, SM inhibits cholesterol transfer by NPC2, even in the presence of anionic lipids. This raised the question of how the cholesterol content itself affects ASM activity toward SM and PC in a detergent-free liposomal assay.

In the following studies, liposomes containing 5–40 mol% cholesterol were used. Liposomes containing less than 5 mol% cholesterol, in particular neutral liposomes, were not included because they are labile and tend to fuse. Their diameters enlarge after two weeks at 4°C up to 10-fold, from 116 ± 20 nm to 1,143 ± 203 nm. Even anionic cholesterol-poor liposomes containing only 6–10% cholesterol enlarge slowly within two weeks (data not shown).

**Fig. 5.**
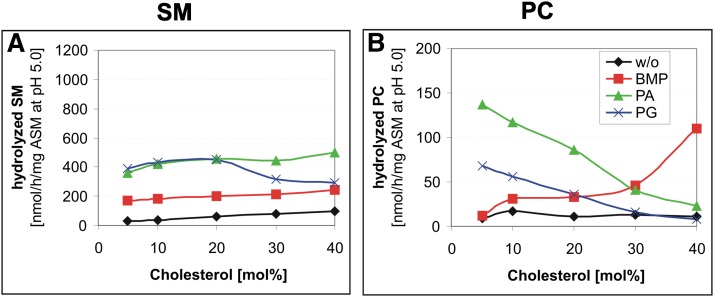
Cholesterol hardly affects SM cleavage, but stimulates PC hydrolysis in the presence of BMP and inhibits it in the presence of PA and PG. ASM activity toward SM (A) and PC (B) was determined with the same assay. Assays were performed in the presence of varying cholesterol concentrations and in the absence (without, black line) or presence of 20 mol% anionic lipid [BMP (red line), PA (green line), PG (blue line)]. Black, neutral liposomes; red, 20 mol% BMP; green, 20 mol% PA; blue, 20 mol% PG containing liposomes. SEM was ± 10%.

Increasing cholesterol concentrations up to 40 mol% induces a slightly higher rate of SM hydrolysis by ASM in neutral and also in anionic BMP- or PA-containing liposomes (**Fig. 5A**). However, in the presence of PG, PI, or PS (PI and PS data not shown) an optimal rate was reached at 20 mol% cholesterol (blue line, Fig. 5A), decreasing again at higher concentrations.

PC hydrolysis by ASM, however, was differently affected by increasing cholesterol concentrations in the presence of anionic phospholipids occurring in cellular membranes (e.g., PA and PG) and BMP, which is generated in the luminal membranes of late endosomes. In the presence of PA and PG, increasing cholesterol concentrations inhibited PC cleavage by ASM substantially, whereas increasing cholesterol levels in the presence of BMP increased PC cleavage up to 8-fold (Fig. 5B).

### DAG and Cer also regulate PC and SM cleavage by ASM

**Fig. 6.**
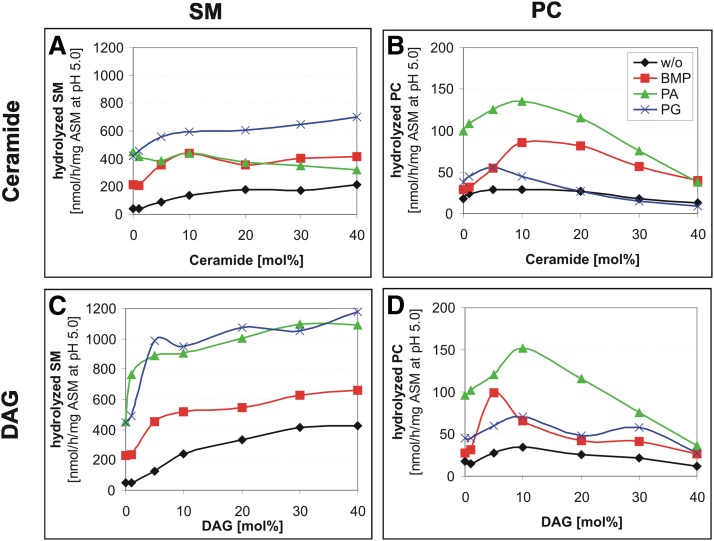
Cer and DAG stimulate the hydrolysis of liposomal SM by ASM and that of PC at low concentrations up to 10 mol%. Cer and DAG stimulate the hydrolysis of SM by ASM (A, C). However, the degradation of PC was upregulated within the same assay only at low Cer and DAG concentrations (B, D). Black, neutral liposomes; red, 20 mol% BMP; green, 20 mol% PA; blue, 20 mol% PG containing liposomes. SEM was in the range of 5–11%.

ASM degrades SM and PC to Cer and DAG, respectively. These reaction products also enhanced ASM activity toward SM and PC at concentrations up to 10 mol%. An increasing Cer content caused a 4.8-fold increase of SM hydrolysis in neutral liposomes and a 2-fold increase in anionic liposomes (**Fig. 6A**). An increasing DAG content, as released from glycerophospholipids by ASM or other phospholipase Cs, triggered SM cleavage by ASM up to 8.7-fold in neutral liposomes and up to 3-fold in negatively charged liposomes (Fig. 6C).

### PLA2 hydrolysis products influence the ASM-mediated cleavage of SM and PC differently

In the late endosomes and lysosomes, most of the phospholipids would be degraded by lysosomal phospholipase A. Therefore, the effect of intermediates of endolysosomal lipid degradation [free fatty acid, lyso-PC, and monoacylglycerol (MAG)] on hydrolysis of SM and PC by ASM was investigated. For these studies, liposomes containing up to 30 mol% free fatty acids, lyso-PC, and MAG were used. At these concentrations, none of the mono-tailed lipids affected the size of the vesicles significantly, as concluded from DLS. Also, no significant formation of micelles was detected.

**Fig. 7.**
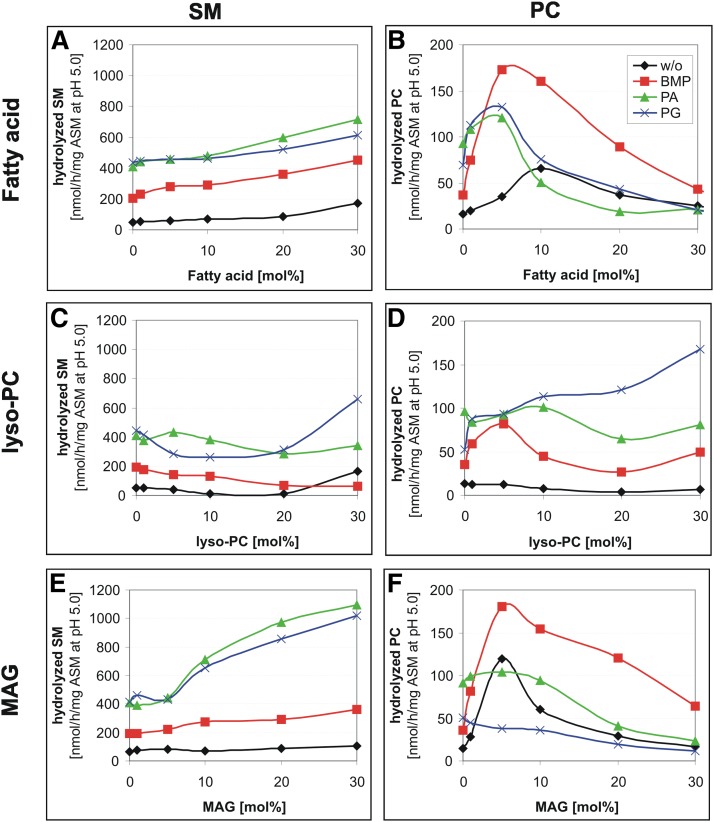
Dependence of SM (A, C, E) and PC (B, D, F) hydrolysis on the concentration of lyso-PC, free fatty acid (palmitic acid), and MAG in liposomal membranes. Experiments were performed as described in Fig. 3. Black, neutral liposomes; red, 20 mol% BMP; green, 20 mol% PA; blue, 20 mol% PG containing liposomes. SEM was in the range of 8–12% (A, C, E) and 15–17% (B, D, F).

Mono-tailed lipids affected SM and PC cleavage by ASM differently. Free fatty acids at concentrations greater than 10 mol% enhanced the degradation rate of SM slightly (**Fig. 7A**) in the absence, as well as in the presence, of anionic lipids (up to 3-fold). The lyso-PC content had no significant influence on the SM and PC hydrolysis by ASM (Fig. 7C, D). Only the addition of PG resulted in an increase of PC hydrolysis up to 3-fold (Fig. 7D), whereas increasing MAG concentrations stimulated SM cleavage up to 3-fold. PC hydrolysis by ASM was enhanced by fatty acid concentrations below 10 mol% up to 4-fold, by lyso-PC only in the presence of PG and by MAG at 5 mol% in the absence of anionic lipids or in the presence of BMP up to 4-fold (Fig. 7B, D, F). High rates of PC hydrolysis by ASM were observed at 5 mol% of BMP in the presence of free fatty acids (Fig. 7B) or MAG (Fig. 7F).

## DISCUSSION

### Membrane lipids regulate membrane-associated proteins

In the last decades, it has been demonstrated that lipids are crucial for cellular functions that are mediated by membranes or take place at membrane surfaces. Membrane lipids play a strong role in the regulation of membrane-associated enzymes and receptors. The individual lipid composition of subcellular membranes and their asymmetric lipid distribution are important for cellular homeostasis and for specific lipid-protein interactions, e.g., the modulation of COP I protein function by SM and cholesterol (47) and the regulation of Na^+^, K^+^-ATPase of plasma membranes by cholesterol (48, 49). Membranes of subcellular organelles keep individual patterns of PI phosphates mediating the interaction with specific cytosolic proteins (50).

The lysosomal catabolism of membrane bound (glyco)sphingolipids such as GM1 (51), GM2 (52), sulfatide (53), sulfated gangliotriaosylceramide (54), glucosylceramide (55), and Cer (56) is strongly stimulated by anionic phospholipids. Dysfunction in the lysosomal sphingolipid digestion leads to lipid accumulation resulting in fatal lipid storage diseases (1). Furthermore, accumulating material affects many lysosomal proteins, thus causing a lysosomal traffic jam (57).

### NPC2-mediated cholesterol transfer is stimulated by ASM-hydrolyzing phospholipids

ASM deficiency in Niemann-Pick disease types A and B causes an accumulation of SM which is accompanied by an increase in lysosomal cholesterol (17). This may well be explained by the inhibition of NPC2-mediated cholesterol transfer by increasing SM levels (7). Restoration of normal ASM activity levels by transfection of NPC1-deficient CHO cells with the *SMPD1* gene resulted in a strong reduction in lysosomal cholesterol levels (58). Indeed, ASM has profound effects on the cholesterol transfer by NPC2. In vitro experiments (Fig. 1) demonstrate that the hindrance of NPC2-mediated intervesicular cholesterol transfer by accumulating SM is overcome by ASM-catalyzed hydrolysis of SM to Cer. This reduces the inhibitory SM levels and generates the stimulatory Cer. This finding can, at least in part, explain the cholesterol accumulation in ASM-deficient patients with Niemann-Pick disease types A and B.

In the ASM-containing experiment given in Fig. 1A, SM content of the liposomal membranes decreased by about 15%. Because ASM can only attack SM in the outer leaflet of the liposomal membrane, its contents should decrease by up to 30%. In an ASM-free experiment (Fig. 1B) containing 8.4 mol% SM (about 30% less than at the start of the ASM-containing experiment) and 1.6 mol% Cer, however, the cholesterol transfer rate by NPC2 reached a significantly lower level (1.8 μmol/h/mg NPC2) (Fig. 1B) than in the ASM-containing experiment (3.2 μmol/h/mg NPC2) (Fig. 1A). Assuming that in the ASM-containing study only the outer leaflet of the liposomal membrane is primarily affected by a decrease of SM and an increase of the Cer level, an asymmetric change in the lipid composition may affect the stability of the lipid bilayer and thereby facilitate recognition and binding of NPC2 to the cholesterol molecules of the liposomal membrane. Because cholesterol can easily flip between both lipid layers of the liposomal membrane (59), this may further speed up intervesicular transfer of cholesterol by NPC2. In addition, and in contrast to the ASM-free experiment of Fig. 1B, ASM may also cleave some PC in the outer leaflet of the liposomes (Figs. 2, 3), further destabilizing the liposomal membranes. However, it cannot be excluded that the ASM protein itself, with its Sap-like domain (60), interacts directly with the outer leaflet of the liposomal membrane, thereby distorting the membrane structure and mediating an increased interaction of NPC2 with the membrane bound cholesterol.

### Cholesterol affects ASM activity toward PC, but not toward SM

Increasing endolysosomal cholesterol levels in Niemann-Pick disease type C, caused by an inherited defect of a steroid transfer protein, either NPC2 or NPC1, are accompanied by an accumulation of SM (61), glycosphingolipids (62, 63), sphingosine (64), and the anionic endolysosomal marker phospholipid, BMP (65, 66). A plausible cause for the lysosomal SM accumulation might be the reduced ASM activity observed in cells and organs of NPC1-deficient patients and mice (58, 67–70). ASM activity might be reduced by proteolytic degradation of ASM protein: accumulating cationic sphingosine in NPC disease may trigger ASM degradation, as observed by cationic desipramine in cell cultures (25, 26). On the other hand, increased levels of the membrane-stabilizing cholesterol in the endolysosomal compartments effectively inhibit sphingolipid activator proteins essential for glycosphingolipid catabolism, e.g., Sap A (71), Sap B (72), and GM2 activator protein (S. Anheuser et al., unpublished observations). Their inhibition could trigger increased levels of gangliosides GM2, GM3, lactosylceramide, and glucosylceramide, even in different vesicles of NPC-deficient cells (73).

Our aim was to test whether the cholesterol content of membranes also affects the ASM activity toward membrane-bound SM and PC in vitro. Surprisingly, it did not affect the ASM activity toward SM degradation (Fig. 5A), but inhibited ASM-catalyzed PC hydrolysis. Increasing cholesterol concentrations reduced PC hydrolysis strongly in the presence of PA and PG (Fig. 5B).

On the other hand, increasing cholesterol levels stimulated PC and also, to a lesser extent, SM cleavage in the presence of BMP. BMP is an anionic lysophospholipid found predominantly in lysosomes and intravesicular membranes of late endosomes (38, 66). As a lyso-lipid, it destabilizes membranes and may enhance the availability of phospholipid substrates for ASM at the liposomal surface. Though this observation is not well-understood, one might expect that admixing membrane lipids with quite different structures, like PG, PA, BMP, and cholesterol, may affect interaction of ASM with membrane bound SM and PC differently. Alternatively, admixed membrane lipids may differ in their affinity to ASM and affect its activity and substrate specificity as potential allosteric regulators (see below).

### Regulation of ASM activity and specificity by lipids of the substrate-carrying membranes

In the present work, the effect of various anionic, neutral, and cationic membrane lipids on the function of ASM toward liposomal phospholipids (Figs. 3, 4; supplementary Fig. I) was studied. Catabolism of complex lipids and other macromolecules is facilitated at low pH values (pH 4–6) in the endolysosomal compartment. Because proteins involved in sphingolipid digestion have isoelectric points at higher pH values, they are protonated at endolysosomal conditions and bind electrostatically, as cationic proteins, to anionic surfaces of the luminal vesicles. A negative surface charge may well be conferred to these vesicles by their presumed content of anionic phospholipids (such as PA, PG, PI, PI phosphates, and sialic acid-containing glycosphingolipids originating from PM and other cellular membranes) initially present before they are degraded. Furthermore, anionic BMP is generated during endocytosis within the luminal membranes as an intermediate of PG catabolism (74, 75).

SM embedded in neutral liposomes was hydrolyzed very slowly by ASM (up to 41 nmol/mg ASM/h), whereas PC catabolism was hardly detectable (Fig. 3A). Incorporation of anionic lipids into the substrate-carrying liposomes, however, increased the hydrolysis rate of both SM and PC by more than 10-fold (Fig. 3), depending on the nature of the anionic phospholipids. Those with an unprotected negative charge at the membrane surface (PA, PG) were more efficient than those having their negative charge covered by a serine or inositol residues. Host lipids containing two unsaturated acyl chains gave the highest catabolic rates, presumably by increasing membrane fluidity (Fig. 2). Furthermore, binding of ASM to liposomal surfaces may also depend on its affinity to individual membrane lipids. For example, PG as a membrane component and a substrate of ASM (10) may compete with other phospholipids (e.g., SM and PC) for binding to ASM differently and more strongly than PS or PI, even though all of them have one negative charge in common.

The incorporation of various anionic phospholipids apparently created a negative surface charge on the substrate-carrying liposomes at pH 5.0, as revealed by measurements of their ζ potential in the range of −26 to −22 mV. On the other hand, ASM, which has an isoelectric point of around 6.8 (20), had a positive ζ potential (4.4 ± 0.2 mV) at pH 5.0, suggesting a positive surface charge. It is conceivable that the cationic ASM is also electrostatically bound to and concentrated at the anionic vesicular surface, allowing the phospholipid degradation at the lipid-water interface, a concept supported by association studies of ASM to liposomes at low pH values (Fig. 4). As expected, the ζ potential of cationic liposomes was positive in the range of 22–24 mV at pH 5.0. Taken together, the electrostatic interaction between ASM and target membranes seems to be an important factor for phospholipid degradation, besides different affinities of ASM to single membrane lipids, and correlates with the ASM activity toward SM and PC (Figs. 3, 4; supplementary Fig. I).

ASM activity is highly stimulated by anionic phospholipids, at least in part by inducing high binding rates of ASM to liposomes, whereas neutral and especially cationic liposomes hardly bind any substantial amounts of ASM. Cationic lipids, which have their net charge localized in the hydrophilic headgroup (see structure in supplementary Fig. IB, C), reduced ASM binding by electrostatic repulsion, and thereby also cleavage of liposomal SM and PC by ASM. In the case of vesicles containing DOTMA, however, the electrostatic repulsion is clearly reduced (Fig. 4). Its positive charge is more located in the lipophilic part of the membrane, which is surrounded by zwitterionic, though neutral, headgroups of PC and SM. This may explain why SM and PC cleavage is still comparable to that in neutral vesicles (Fig. 3A, J; supplementary Fig. IA, D).

Previous studies have shown that lipid cleaving activity of several endolysosomal hydrolases is stimulated more by PA and BMP than by PS and PI [arylsulfatase A (53), lysosomal phospholipase A2 (76), acid glucocerebrosidase (55)]. Also, lipid mobilization and solubilization by Sap B are higher in liposomes containing PA than in those containing BMP at low pH (72). The reduced electrostatic interaction of PI and PS could be due to steric shielding of their negative charge by inositol and serine residues, respectively.

**Fig. 8.**
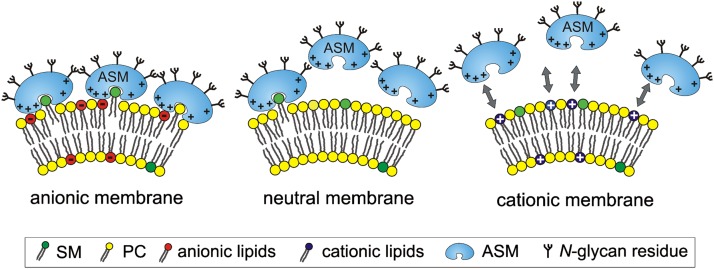
Model of the electrostatic interaction of ASM with anionic, neutral, and cationic membranes at low Ph. +, positively charged; −, negatively charged; green, SM; yellow, PC; red, anionic lipid (e.g., BMP, PG, PA); blue, cationic lipid; gray arrows, electrostatic repulsion.

As previously observed, electrostatic binding plays an important role in the interaction between proteins and charged lipids at membrane surfaces (**Fig. 8**) (25, 77).

The uptake of added cationic amphiphilic drugs, like desipramine (25, 26) or amitriptylin (78), by cultured fibroblasts and their incorporation in luminal lysosomal vesicles may also explain the release of ASM from intraendolysosomal vesicular surfaces, followed by proteolytic digestion, as suggested by in vitro and cell culture experiments (25, 26).

### Products of lysosomal hydrolysis generated by ASM and phospholipase A stimulate SM catabolism

Previous studies have shown that ASM purified from human tissue does not only hydrolyze SM, but also hydrolyzes various glycerophospholipids like PC and PG at a low rate (10, 45, 46). These studies could not rule out, however, that the low hydrolysis rates of various glycerophospholipids were due to a contaminating phospholipase C. It is shown here that the highly purified recombinant human ASM also splits PC. The ASM is, however, 11-fold more active on liposomal SM than toward PC (Fig. 2). Interestingly, the specificity for SM and PC hydrolysis is also substantially modulated by membrane lipids. Increasing levels of DAG, fatty acids, MAG, and Cer enhance SM hydrolysis, whereas PC cleavage usually passes through an optimum at around 10 mol% of the lipids, with higher concentrations being inhibitory. In previous studies, DAG has already been described as a stimulator for ASM activity in micellar and/or detergent-containing nonliposomal assays (79–81).

ASM prefers SM as substrate, especially in the presence of cholesterol, conditions that may prevail in early endosomes. However, PC may also become a suitable substrate during endocytosis in late endosomes, when low cholesterol levels are reached in the presence of increasing BMP levels. Important questions, however, remain unanswered: How long can vesicle and membrane structures be maintained during phospholipid degradation by ASM (SM, PC) and phospholipase A (glycerophospholipids)? Vesicular membranes should become leaky and the topology of luminal vesicles should eventually break down during phospholipid and sphingolipid degradation. Membrane structures of the luminal vesicles of the lysosomes will disappear and may end-up in lipophilic lipid-protein aggregates, in micellar or unknown structures.

## CONCLUSION

**Fig. 9.**
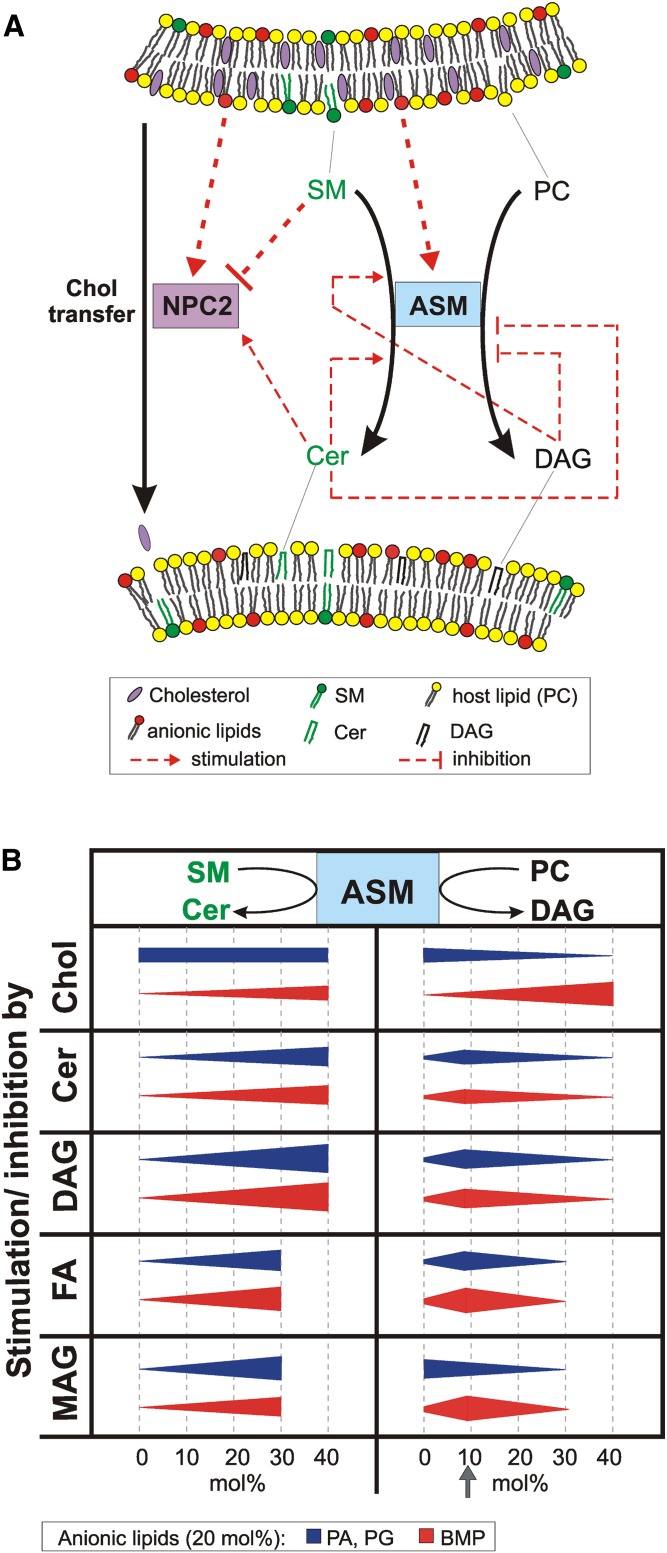
Membrane lipids regulate ASM activity and cholesterol transfer by NPC2. A: Model for endolysosomal lipid degradation and cholesterol transfer between vesicular membranes. B: Illustrates the effect of different membrane lipid on the ASM activity toward liposomal SM and PC.

Membranes are composites of proteins, phospholipids, glycosphingolipids, and cholesterol. During endocytosis, indigestible cholesterol is sorted out and the other membrane components are degraded at luminal membranes of the endolysosomal compartment. Our data suggest that the digestion of SM by ASM is crucial for the endolysosomal lipid degradation and sorting. The conversion of SM to Cer stimulates the cholesterol export by NPC2 (Fig. 1). Its regulation by membrane lipids and the regulatory impact of different lipids on the NPC2-mediated cholesterol transfer is summarized in **Fig. 9**. Though, cholesterol does not affect the ASM activity toward SM, high cholesterol levels inhibit lysosomal glycosphingolipid catabolism at several points, even in the presence of stimulatory anionic lipids, e.g., the function of lipid transfer proteins Sap A (71), Sap B (72), and GM2AP (S. Anheuser et al., unpublished observations), which are essential for glycosphingolipid catabolism.

## Supplementary Material

Supplemental Data
