# Supplementary material for: Acid sphingomyelinase activity is regulated by membrane lipids and facilitates cholesterol transfer by NPC2
Source: J Lipid Res. 2014 Dec;55(12):2606–19. doi: 10.1194/jlr.M054528 (PMC4242453; doi:10.1194/jlr.M054528)
Supplement: Supplemental Data [file supp_55_12_2606__index.html]

Acid sphingomyelinase activity is regulated by membrane lipids and facilitates cholesterol transfer by NPC2 — Acid sphingomyelinase activity is regulated by membrane lipids and facilitates cholesterol transfer by NPC2 — Supplemental Data 

# Acid sphingomyelinase activity is regulated by membrane lipids and facilitates cholesterol transfer by NPC2

## Supplemental Data

**Files in this Data Supplement:**

- Supplemental Fig 1 - Supplemental Fig 1
