# Supplementary material for: Acid sphingomyelinase activity is regulated by membrane lipids and facilitates cholesterol transfer by NPC2
Source: J Lipid Res. 2014 Dec;55(12):2606–19. doi: 10.1194/jlr.M054528 (PMC4242453; doi:10.1194/jlr.M054528)
Supplement: Supplemental Data [file supp_M054528_jlr.M054528-1.pdf]

Supplemental Data

Supplementary Figure 1:

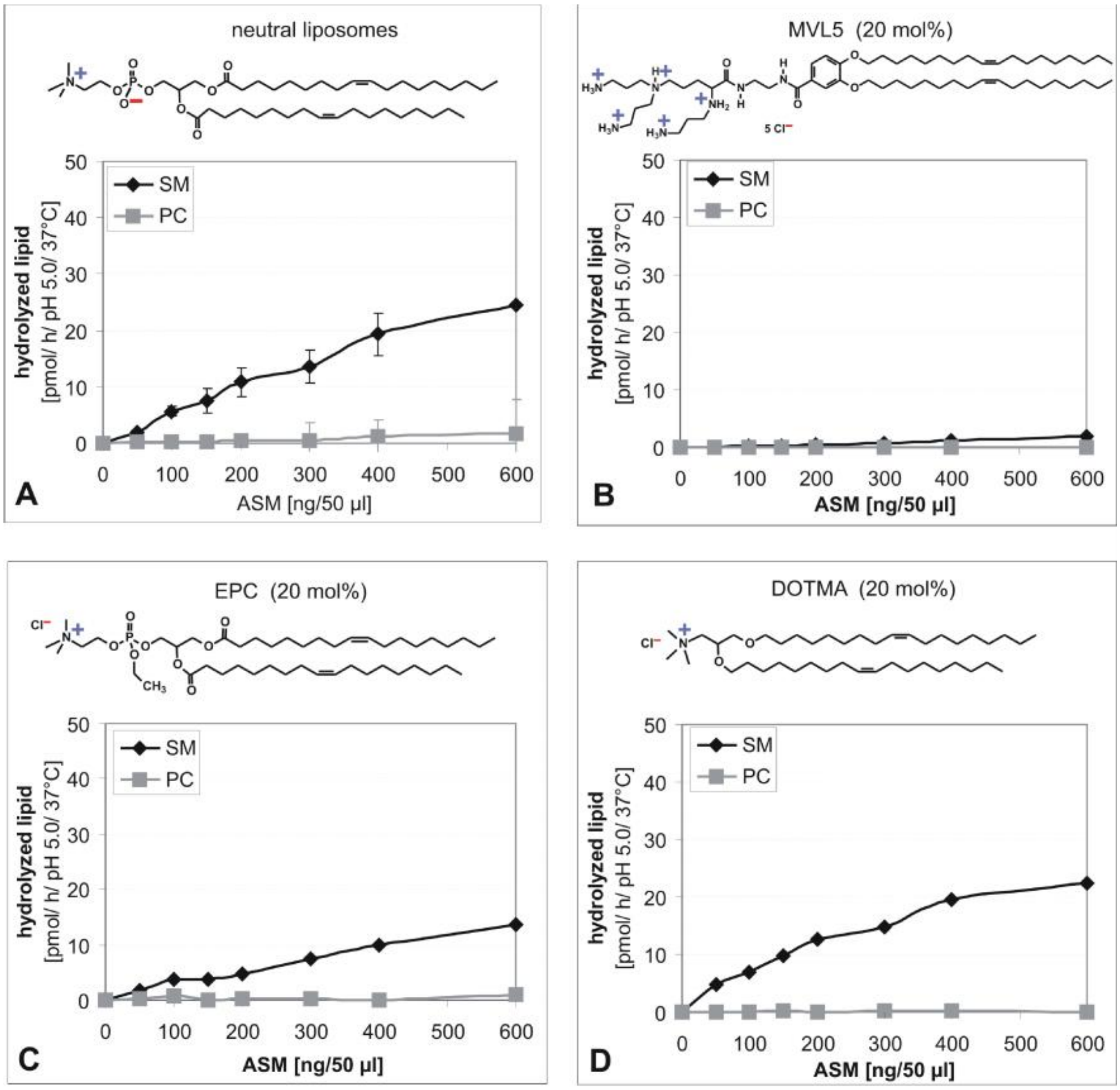

Supplementary Fig. 1: Cationic membrane lipids inhibit the degradation of liposomal SM and PC by ASM at low pH

Hydrolysis of SM (black line) and PC (gray line) was assayed at pH 5.0 at different ASM concentrations using neutral and cationic liposomes as carriers for the substrates, SM and PC. Positively charged liposomes generally consisted of 60 mol% DO-PC, 20 mol% cationic lipid (MVL5 (**B**), EPC (**C**), DOMTA (**D**), 10 mol% cholesterol and 10 mol% SM.

DOMTA: 1,2-di-O-octadicanyl-3-trimethylammonium propane; EPC: O-ethyl phosphatidylcholine; MVL5: multivalent cationic lipid. Error bars indicate SEM (n=4)

Fusion assay

In the fusion assay two types of liposome were used. Liposome type I contained 10 mol% cholesterol, 20 mol% BMP, 66 mol% DO-PC, and **4 mol% Biotin-PE**. The type II liposomes consisted of 10 mol% cholesterol, 20 mol% BMP, 65.5 mol% DO-PC, **0.5 mol% [^{14}C] PC**, and **4 mol% NBD-PE**. The final lipid concentration of each liposome preparation was 200 nmol/ml in 20 mM citrate buffer (pH 5.0), containing 150 mM NaCl. Both types (type I and II) of liposomes (4 nmol in 20 µl each) were incubated together with 150 ng ASM in a final volume of 200 µl for 10 min at 37°C. The assay was stopped by adding 75 µl of 1 M Tris buffer (pH 8). To separate the unfused liposomes I and the fused liposomes of type I and II containing Biotin-PE , streptavidin-coated paramagnetic beads (BioMag® Streptavidin) were added. After 10 min incubation at room temperature the Biotin-PE-containing vesicles were pulled to one side of the tube wall using a magnetic separation stand. The unfused liposome II, containing the marker lipids [^{14}C] PC and NBD-PE, remain in the supernatant. Aliquots of the supernatant were measured for radioactivity by scintillation counting (100 µl of supernatant) and for the fluorescence (150 µl supernatant diluted with 250 µl water) by RF-5000 spectrofluorimeter (Shimadzu, Kyoto, Japan) at an excitation wavelength of 466 nm, emission wavelength of 526 nm.
